# Ecological-environmental challenges and restoration of aquatic ecosystems of the Middle-Eastern

**DOI:** 10.1038/s41598-022-21465-0

**Published:** 2022-10-14

**Authors:** Ahmad Reza Pirali zefrehei, Mahdi Kolahi, Judith Fisher

**Affiliations:** 1 Faculty of Natural Resources and Environment, Ferdowsi University of Mashhad, Mashhad, Iran; 2 Faculty of Natural Resources and Environment, Water and Environment Research Institute, Ferdowsi University of Mashhad, Mashhad, PO Box 9177948974, Iran; 3 Institute of Agriculture, University of Western Australia, 35 Stirling Highway Crawley, Perth, 6009 Australia; 4 Fisher Research Pty Ltd, PO Box 169, Floreat, Perth, 6014 Australia

**Keywords:** Environmental sciences, Environmental social sciences

## Abstract

Water resource management has numerous environmental challenges, especially in aquatic ecosystems such as rivers due to the heterogeneous distribution of surface water resources, among other diverse impacting factors. In Iran (one of the countries of the Middle-East), population growth, development of urban communities and development of agricultural and industrial activities provide additional impacts on the functioning of aquatic ecosystems. The United Nations declared the third decade of this century (2021–2030) as the decade of ecosystem restoration. In this study, we have selected the Zayandehroud River as a case study and then evaluated the pathology of existing statuses. Strategies and approaches were studied and analyzed including the need to utilize integrated water resources management (IWRM), approaches for dealing with drought conditions, payment of water rights and dam alternatives, and the need for ecological landscape studies. Then, strategies and approaches appropriate from the perspective of restoration were identified, including the techniques used, and the experiences of different countries. The analysis showed that similar regions of Iran in the Middle-East need to change the paradigm of "nature control" to the paradigm of "nature management" and reduce reliance on structural and technological solutions in water resources management.

## Introduction

Iran is one of the driest countries in the world with an average rainfall of 240 mm per year and has a heterogeneous spatial distribution. 1% of Iran's area has more than 1000 mm of rainfall, while 28% of the country has less than 100 mm of annual rainfall. Approximately 70% is evaporated of the 415 billion cubic meters of annual precipitation in Iran^1^. According to existing definitions, regions with less than 1000 m^3^ of water available per person per year have unsuitable conations. The continuation of the current trend of population growth, lack of proper water demand management and reform of water consumption patterns will lead to Iran face a serious water crisis in the near future. By 2050 Iran is expected to be among the water-scarce countries^2^. Madani et al. have addressed the main problems associated with Iran’s water management and have identified some of the challenges, including, "rapid population growth, migration and urbanization, inadequate water distribution infrastructure, water quality degradation, inefficient agriculture, the dream of self-sufficiency in food, rising water demand, cheap water and energy, dams, deep wells, droughts, climate change”^3^. In this regard, aquatic ecosystems in the Middle-Eastern are naturally in a critical situation. Table 1 provides some watershed characteristics across Iran. Experts believe that a country's livelihood, prosperity and power are directly dependent on their river ecosystems^4^. The increasing development of urbanization and the transformation of rural areas into cities (this is assuming that these cities have an associated river system) is always accompanied by land use changes and pressure on the river system. Hydrological and morphological changes following urbanization, and their consequences (habitat degradation and reduction of aquatic biodiversity) are summarized in the term "urban stream syndrome"^5,6^. Human pressures affecting the freshwater systems of urban rivers have increased in contrast to non-urban rivers. This is due to the combined effect of multiple pressures, such as water abstraction, fragmentation, canalization, change of coastal vegetation and inland habitats, increase of impermeable surfaces and loss of vertical cohesion^7,8^. The heterogeneous distribution of surface water resources in Iran, and population growth and development of urban communities, and development of agricultural and industrial activities on the other hand, has highlighted the problem of water shortages for drinking, agriculture and industry in some parts of the country. Due to these shortcomings the water supply required for these activities is one of the most important concerns of managers^9^. Therefore, in these cases, water transfer projects are inevitably proposed. One of the most important effects of these types of projects is the adverse effects on the environment and hydrology of the project area of origin. Considering the designation of the decade (2021–2030) as the decade of ecosystem restoration by the United Nations General Assembly, this study intends to examine the existing challenges of aquatic ecosystems in the Middle-Eastern (looking at the Iran and the Zayandehroud River as a case study) and pay attention to providing restoration solutions to deal with these.

**Table 1:** Iran watershed characteristics(ha)^55^.

Basin	Total area (km^2^)	As% of total area	Rainfall (mm/year)	As% of total rainfall
Persian Gulf and Oman Sea	424 209	26	380	39
Orumie Lake	51,801	3	347	5
Caspian Sea	175,051	11	423	18
Hamoon Lake	103,169	6	107	3
Central Plateau	824,356	51	166	33
Qara-Qum	44,165	3	226	2
Total	1,622,751	100	253	100

## Ecosystems and the environment of Zayandehroud River

**Figure 1:**
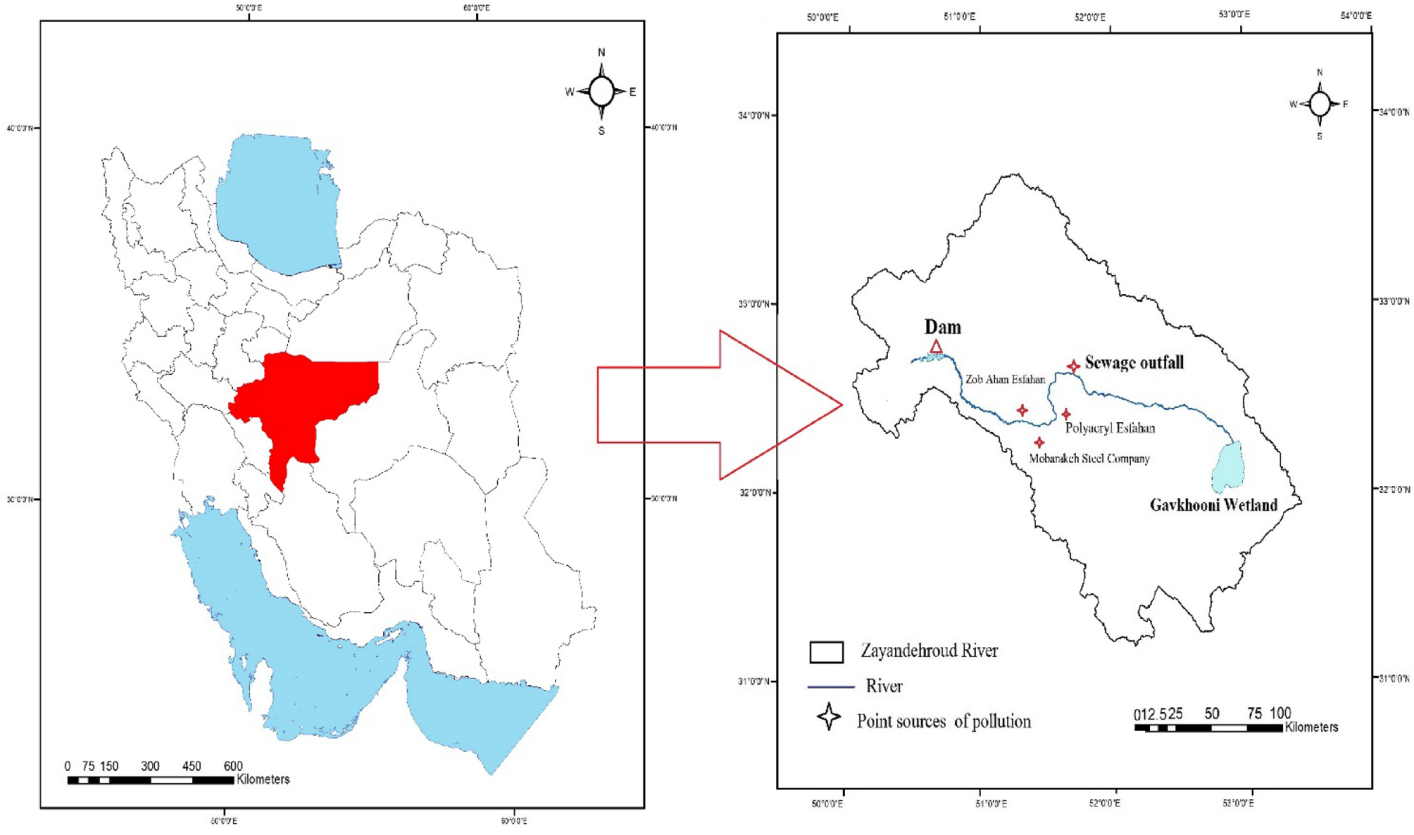
Location of the Zayandehroud River Basin and dam.

The Zayandehroud is one of the important rivers located in the center of Iran. This river originates from the heights of Zagros in Chaharmahal and Bakhtiari province and flows into the Gavkhooni wetland in the southeast of Isfahan. The water from this river is widely used in agriculture, industry and for drinking water. But the influx of agricultural and industrial effluents and municipal sewage into the river places it in an unusual situation. These unusual conditions can be seen downstream of the river. The Zayandehroud River is one river that has undergone many changes due to its location in a special climatic situation (hot and dry region of the Central Plateau of Iran)^10^. (Fig. 1).

**Figure 2:**
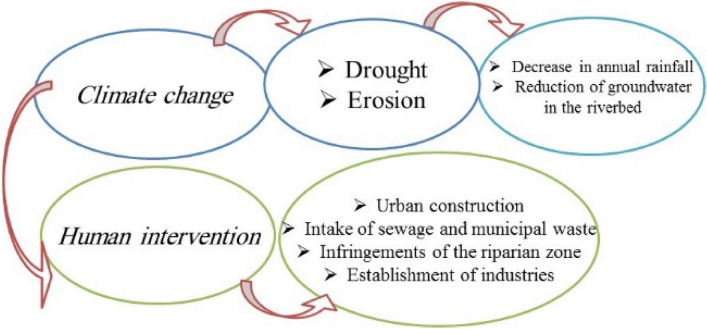
Damages and environmental challenges of the Zayandehroud river.

The upstream of the Zayandehroud basin has faced high levels of drought in recent years. These droughts have led to damaging effects on the river’s hydrology, environment, economy and society. The most important consequences of these droughts have been a reduction in water volume in the Zayandehroud dam and river's flow, severe water table decline, desertification of a large part of the agricultural lands, restriction of industrial activities, and especially the impacts on tourism. Analyzing the nature of the structure of these problems is one of the main concerns of the people, the government and the traditional exploiters of this watershed. These groups have considered a wide range of factors such as management, inadequate abstraction of water resources, expansion of the Zayandehroud watershed beyond its natural watershed, climate change and successive droughts. The damages and challenges to the Zayandehroud River can be divided into two dimensions: climatic effects and human interventions. Figure 2 shows some of the damage and challenges this basin faces. It should be noted that most of these cases can also be seen in similar aquatic ecosystems in Iran.

## Ecological-environmental challenges of aquatic ecosystems

In this section, the challenges are introduced and then analyzed:

### Drought

**Figure 3:**
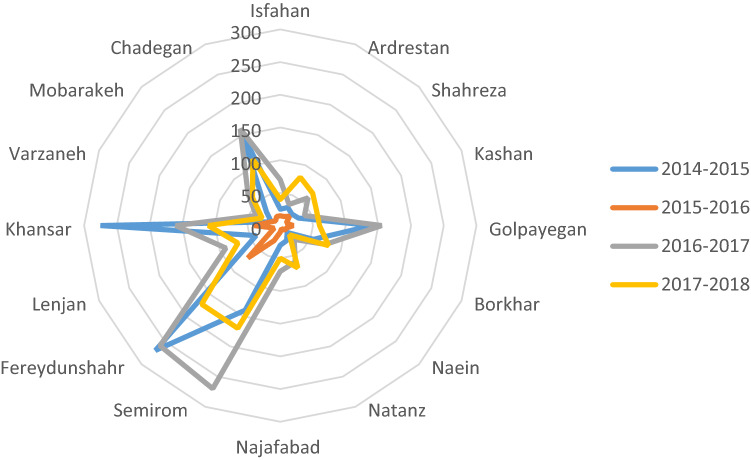
Average rainfall (mm) in different cities of the Zayandehroud basin (2014–2018).

Drought and changes hydrological conditions will have significant effects on the ecological characteristics and biological communities of rivers. The direct effects of drought include reduced water levels and reduced access to habitat for aquatic animals. Its indirect effects include increased inter-species competition and changes in existing natural food resources^11,12^. The gradual drying of rivers over time provides an opportunity for the behavioral adaptations of aquatic organisms. These stresses force many invertebrates to evolve their life cycle or change their behavioral characteristics to increase their survival^11,13^. The Zayandehroud River originates from a cold and mountainous region, but a significant part of its route is located in arid areas and it finally leads to the Gavkhooni wetland located in an arid and desert region. Cities, villages and farms are located on the banks of this river and people’s lives depend on the water of the Zayandehroud River. The dynamism and life in the Zayandehroud valley are strongly dependent on this water flow. Figure 3 shows the changing rainfall in mm between 2014 to 2018 at the key cities within the river basin.

### Construction of a dam and unfair distribution of water rights

**Figure 4:**
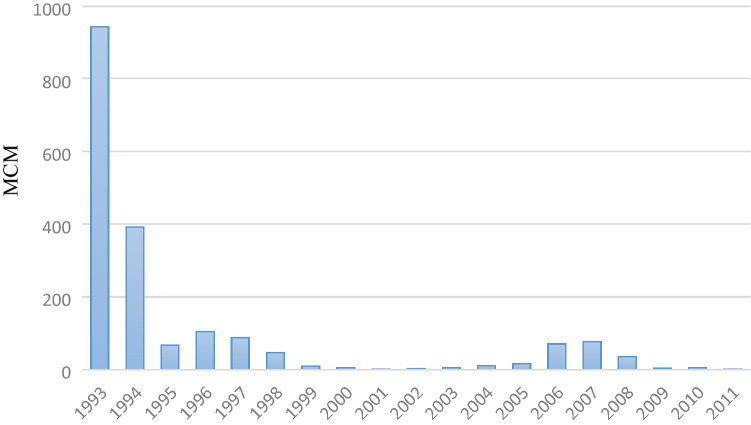
The volume of water entering the Gavkhooni wetland at the end of the Zayandehroud basin (MCM) (1993–2011)**.**

The arid climate of countries such as Iran has led to the construction of dams as an engineering solution, with the use of new technology to regulate the flow of water for crops, drinking, and other uses^14^^.^ Studies conducted on various dams in Iran show that the construction of dams in the country has often not been economically viable. The negative environmental effects of dam construction cannot be easily ignored. In Iran, the Environmental Impact Assessment (EIA) law has been added to the feasibility studies to justify construction of dam^15^. The construction of the Zayandehroud dam in 1970s caused changes to the inflow and water rights of Zayandehroud so that the life of the Gavkhooni wetland at the end of this basin is now experiencing serious threats. In Fig. 4, you can see the amount of water entering the Gakhooni wetland. The catchment area of the Zayandehroud is 3200 km^2^. The most important surface waters of this basin have been regulated by creating the Zayandehroud dam. This dam is located at 2100 m above sea level and 110 km northwest of Isfahan.

### Urban development and establishment of new land uses in the riparian zone

Today, the consequences of the rapid growth of urbanization and urban development, especially in developing countries, has led to experts and planners placing considerable attention on sustainable urban development. Sustainable urban development requires success in achieving quality urban goals and providing conditions for equality of present and future generations^16^. Often, issues overlook the impacts of human activities and land use allocations which take place alongside the river systems in the cities of developing countries. Significantly, the water body is altered by direct and indirect land use, including agriculture, trade, industry, and residential buildings. There are also other side effects on human activities such as direct deposition of solid, liquid, and sand wastes and inappropriate agricultural practices, including improper waste disposal and chemical fertilizers used in agriculture^4^.

The Zayandehroud River has also been influenced by urban development, population growth, establishment of various industries in its margin, and especially different agricultural uses from upstream to downstream (Fig. 1 shows some of the sources threatening the river). On the other hand, the wastewater of Isfahan residents is discharged into the Zayandehroud River after incomplete treatment. In addition, from the city of Isfahan onwards, only agricultural effluent enters the river. However, due to the increasing pressures on aquatic and river ecosystems and the ecological changes caused by these pressures, we are witnessing more and more simplification of the natural systems and changes in their performance and functionality, thus reducing biodiversity. Finally, we see rapid landscape changes in these ecosystems. With changing landscape patterns numerous environmental problems such as soil and water pollution and climate change at the local and regional scales result in the reduction of biodiversity^17,18^. Therefore, studies on these changes require an interdisciplinary approach that broadens the connections between the social sciences, and the natural sciences. This new approach requires an understanding of the interactions between living and non-living components of river systems. An approach that, while considering traditional, economic, social, and political factors, is also based on geological and climatic factors. It is now well established that successful management of natural ecosystems depends on management's awareness of threatening processes, patterns and environmental conditions. It requires better management of resources to understand ecosystem performance and accurate and sufficient planning at the landscape level^19,20^.

In addition, understanding how urbanization affects vegetation in coastal areas of aquatic ecosystems is essential. Because they provide vital ecosystem services and maintain a high level of biodiversity^21^. Coastal regions are strongly affected by urbanization. Urbanization in these areas increases impenetrable surfaces, leading to the destruction of the primary coastal habitat^22,23^. Urbanization and impervious surfaces will reduce the richness, diversity, density and biomass, as well as changes in fish population structure and nutritional structure. One of the most important disorders caused by urbanization on coastal areas is the destruction of vegetation and soil compaction^24,25^. These factors have a significant effect on the hydro-morphological variables of inland habitats and can lead to a decrease in river aquatic richness.

## Strategies and approaches

In this section, strategies and approaches for each of the above challenges are analyzed providing suggestions for future research.

### The necessity of integrated water resources management (IWRM)

At the World Summit on Sustainable Development, the concept of Integrated Water Resources Management (IWRM) was developed as an essential international solution for managing stressful water resources. The accepted concept of integrated water resources management based on the definition of The Global Water Partnership Network (GWP) is as follows: "Integrated Water Resources Management (IWARM) is a process which promotes the coordinated development and management of water, land and related resources, in order to maximize the resultant economic and social welfare in an equitable manner without compromising the sustainability of vital ecosystems”^26^.

Integrated water resources management is aimed at ensuring the sustainable use of water resources. Integrated water resources management is the only comprehensive solution for actions such as reducing traditional water consumption, imposing restrictions on the quantity and quality of water consumption, and making changes in demographic and production patterns to achieve sustainable development. This method provides more transparent management of water resources, the functions and the impact of actions, and can be evaluated and judged incorporating the effect of all components. In this management method, although the planning is done at the macro level and takes into account all the views and needs of different structures and goals, with each department responsible for implementing its area. Therefore, the issue of overlapping responsibilities will not be raised. Executive organizations will work with more confidence in how they work and interact with the performance of other organizations. Integrated management has the potential to dramatically accelerate and enhance the comprehensive water planning process^26,27^. Studies in this field have been carried out which can be referred to Gohari et al.^28^ in the Zayandehroud River. They suggested that factors such as infrastructure improvements, careful water demand management, and ecosystem-based regulatory prioritization could temporarily reduce water stress in this basin.

### Approaches to dealing with drought conditions

**Figure 5:**
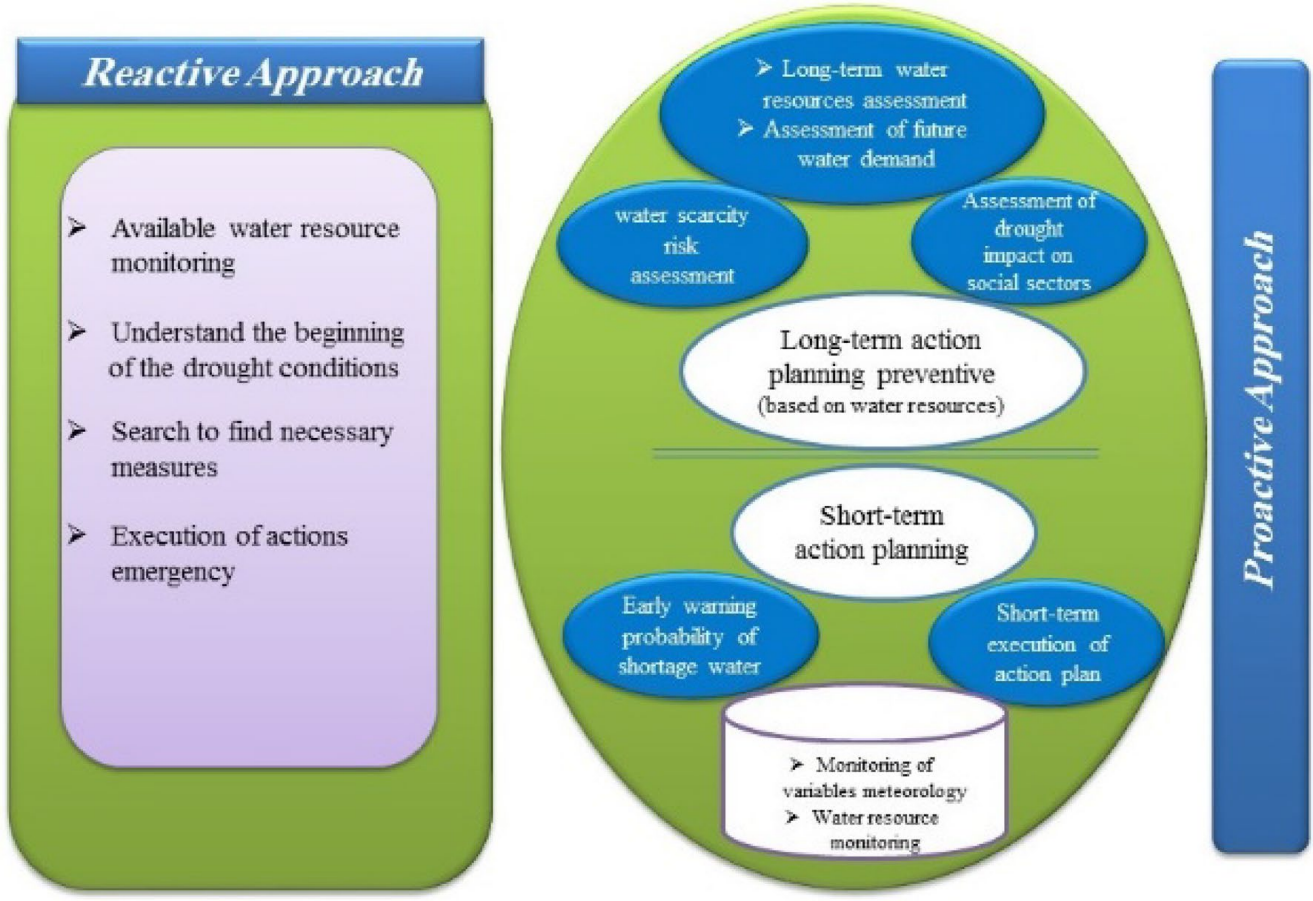
Flowchart of reactive and proactive approaches.

Two approaches have been proposed to deal with drought conditions, which are: (a) Reactive approach: In this approach, as soon as the drought conditions occur and it is understood, efforts are made to find appropriate solutions and to apply them. In this approach, no program is predetermined and the proposed programs are identified and implemented with a delay. (b) Proactive approach: In this approach, managers and decision-makers working in the fields of drought and water have a long-term and predetermined plan, including regular monitoring of water resources and changes in the environment, and as soon as they realize the slightest deviations they proceed to perform and direct predetermined programs^29,30^. Of course, with a closer look at these two approaches it can be seen that a proactive approach to control and manage drought is always the best approach. A summary of the two approaches is given in the flowchart below (Fig. 5).

At the global level tackling drought was one of the critical issues that was raised at the 1992 Development and the Environment conference in Brazil. One of the documents of this conference^31^ referred to the effects of desertification that threatens land droughts. Accordingly, in addition to establishing or strengthening national systems for gathering information and examining the social and economic effects of drought and desertification, governments were also asked to consider the following strategies^31,32^: Applying sustainable land application policies, applying sustainable management on existing water resources, adapting agricultural activities to the environment, using clean technology (with less pollution), protecting existing forests and creation of drought-resistant forests, and combining research activities with indigenous knowledge related to forests and natural vegetation*.* A new report from the United Nations Convention to Combat Desertification (UNCCD), titled "Drought in Numbers 2022" beyond restoration calls a full global commitment to drought preparedness and resilience in all regions of the world^33^. The report added there is a need for a paradigm shift from reactive and crisis-based approaches to preventive and risk-based drought management approaches^33^.

### Payment of water rights and alternatives to dams

Considering that one of the most important factors in the drying of rivers is the construction of deviation clauses and dams, and digging deep wells upstream of them, aligned with this proper management should be given to the allocation water rights. Also, the permitted rate of abstraction from water resources must always follow the law of nature and not human needs for water. Unfortunately, this basic rule has been ignored in the exploitation of water resources in the catchment area of aquatic ecosystems. Although dams have many benefits, including water storage and flood control, they will quickly have negative consequences for river and animal species, ecosystems, water quality, people and livelihoods. Therefore, over time the removal of the dam will be one of the options for the authorities to rehabilitate the river habitat. The option of removing the dam should be presented to the authorities.

Removal of the dam is an effective mechanism to accelerate the restoration of river habitat. It is also an environmentally friendly method of restoring the river ecosystem to its natural regime, which will have advantages and disadvantages. In this context, it is often necessary to examine multiple hydrological, environmental, and social criteria. In the United States, river restoration is one of the main goals of removal of dam projects. Dam removal accelerated in the last three decades of the twentieth century, with more than 200 dams destroyed^34^. Proposals to restore rivers by the removal of dams requires extensive discussion and teamwork covering a wide range of issues. According to the river restoration approach, dams often disrupt the structure and function of river ecosystems by changing flow regimes, disrupting sediment transport, changing water quality, and disrupting their biological continuity. On the other hand, removing the dam can cause significant changes in the processes and morphology of the river canal, the effects of which can be studied^35,36^. An essential aspect of dam removal is planning in the early stages and identifying alternative options (such as hydroelectricity, irrigation, and water supply). Dismantling the dam requires balancing the various roles of the river. Having a comprehensive management plan and the inclusion of alternatives that meet the goals of the dam can minimize the negative effects of dismantling the dam. Dam-free solutions are in line with some of the provisions of the Iranian Constitution and the country's international obligations, and are more in line with sustainable development practices. The private sector can cooperate well in this approach, and of course, these solutions are less expensive than building a dam.

## The necessity of landscape ecology studies

Understanding the region's landscape and applying the principles and concepts of ecology, biodiversity protection and sustainable development of the river ecosystem is understood more and more due to the comprehensiveness of landscape ecology studies. In this section due to the lack of such information in areas similar to Iran, we will introduce and explain the principles of landscape ecology in a river ecosystem. The river corridor is part of the landscape where the focus is on the existence of water^37^. The concept of river landscape was proposed in 1960s, following its use by Leopold and Marchand to describe rivers on a large scale of physical, biological, and natural beauty^38^. However, it did not receive much attention until the early 2000s. In the concept and perspective of patch dynamics across the landscape, the river system is considered as a combination of large-scale patterns of energy, materials, habitat structure, differentiation in local zones and patches^38^. In recent decades, the study of river systems management with the river landscape design approach has become evident^37,39^. Structure, function and dynamics are the three main features of Landscape ecology studies^40^.

Today, proper management of aquatic and terrestrial ecosystems requires a correct understanding of all landscape features and the processes between them. The nature of a landscape is defined by its structural elements, namely matrix, patch, and corridors, and the recognition of these elements depends on the concept of spatial and temporal scale.

The set of patches forms a mosaic, and the set of corridors forms a network. The spatial arrangement of mosaics and grids forms the landscape model, and the landscape is structurally distinguished by these patterns. By identifying the landscape elements, the relationships between them, and changing these relationships over time, it is possible to make better decisions about managing and directing the landscape change process. In this regard, landscape ecology is a science that studies the spatial pattern of ecosystems, how these patterns change and how they interact over time^37,39,40^.

## Environmental impact assessment

Since the end of 1960, Environmental Impact Assessment has been introduced as an activity to identify and predict the effects of development measures on human welfare, health, the bio-geophysical environment, and to study and disseminate information on these effects. Its legal implementation has found a special place in different countries of the world^41^. In Iran, this began in 1975 with the introduction of regulations related to environmental impact assessment and in 1994 it became completely legal. The most important purpose of conducting EIA as a tool for environmental management is to ensure compliance with the policies and objectives set out in the plans and activities of a project in line with the rules, criteria, laws, and regulations of the government to be able to provide options by improving the quality of the human environment and to prevent the development of pollution and destruction of nature by development projects. Management practices in protecting the environment of rivers and reducing the destructive environmental effects in the restoration of urban rivers require planning and solutions for administrative measures that require the use of sufficient knowledge in the study and analysis of related factors. Bond and Dusík examined the impact assessment process in the face of global challenges associated with climate change, the fourth industrial revolution. They presented approaches including formalizing technology assessment processes and/or the inclusion of emerging technologies within the scope of legislated IA processes^42^.

## Restoration of aquatic ecosystems

Before considering and analyzing restoration of aquatic ecosystems as a strategy and approach, the relevant terms should be introduced^43,44^. Then we analyze this approach.

### Restoration

The ideal project for river restoration is to restore the river to its natural and basic conditions, which includes water quality, sediments, flow regime, canal geometry, local aquatic plants and organisms.

### Rehabilitation

Although complete restoration may be impossible, rehabilitation can bring the destroyed river system to promising conditions by improving important environmental aspects of the river.

### Remediation (replacement, reclamation)

In some cases, even rehabilitation is not possible due to irreversible river changes. In such circumstances, it can often be said that the baseline conditions are no longer a suitable target for that river. The goal of remediation is to improve the ecological condition of the river, but the final condition of this improvement does not necessarily recreate the basal condition of the river.

Restoration of rivers is defined as: “Assisting the recovery of ecological structure and function in a degraded river ecosystem by replacing lost, damaged or compromised elements and re-establishing the processes necessary to support the natural ecosystem and to improve the ecosystem services it provides”^43^.

**Figure 6:**
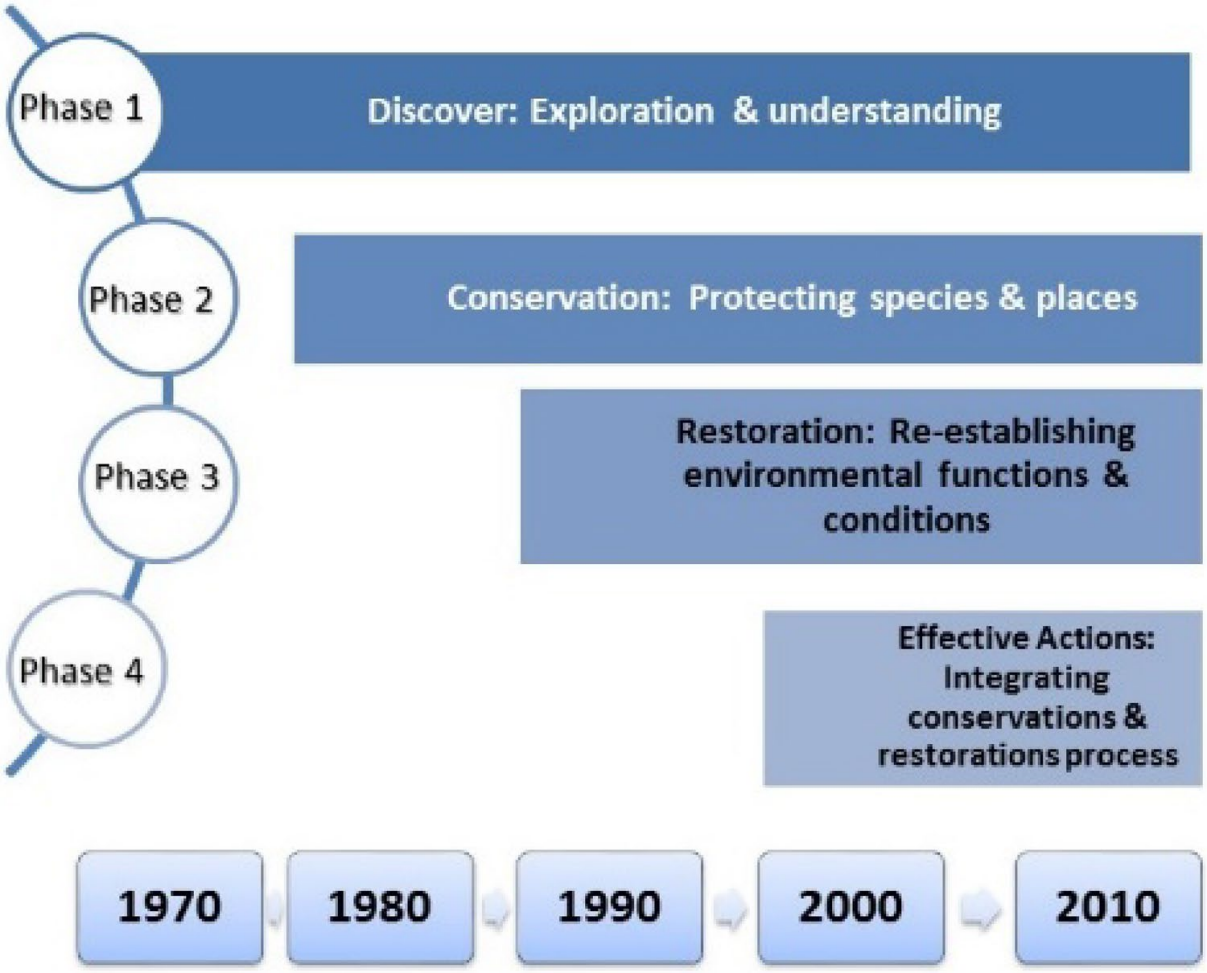
Four phases in the evolution of river conservation and restoration.

With the emergence of economic, social, and environmental consequences of human interventions in ecosystems, "sustainable development" gradually emerged in the world community in the last three decades of the twentieth century. The United Nations Conference on the Environment and Human Rights in Stockholm (1972), the Cocoyoc Declaration (1974), and finally, the Earth Summit in Rio de Janeiro (1992) illustrate this trend. This attitude has affected all areas of human activity on the biosphere; including the traditional paradigm of water resources management, which has been criticized for its continuous exploitation of nature. Newer paradigms such as Integrated Water Resources Management (IWRM) and Adaptive Management have taken a more holistic and friendly approach to ecosystems. Simultaneously with this trend, the "river restoration" approach to water and environmental management has been growing since the beginning of the twenty-first century. Figure 6 shows a schematic diagram of the evolution of river restoration^45^.

## Challenges facing the restoration of surface water resources in Iran

The most critical challenges facing the restoration and rehabilitation of water bodies in Iran are as follows:

Lack of national determination to protect the environment; In recent years there have been widespread discussion in the country about sustainable development and the relationship between health and economic and social growth of human society however a healthy and dynamic environment. However so far there is no public determination to pay attention to the needs of the environment among people and officials and the importance of returning water resources to normal conditions.

Lack of centralized management of water resources and water bodies; In many developed countries, the management of water resources and the environment of water bodies is concentrated and these two are considered as a single set. In Iran, the management of the country's water resources and the environment of water bodies are in two separate groups and are ultimately cross-sectoral issues.

Sewage entering water bodies; One of the most severe current problems of Iran water management and the department of environment (DOE) is the lack of extensive facilities for wastewater treatment in the industrial and agricultural sectors, which in many cases are discharged to surface water sources. This has irreversible effects on the aquatic ecosystem (for example, the discharge of wastewater into the Karun River and the establishment of a wastewater treatment plant downstream of the Zayandehroud River along with entry of agricultural effluent in its different parts).

Uncertainty of the role of stakeholders in the preservation of water bodies; Water bodies belong to everyone as a national treasure and there must be public participation in maintaining their conditions. However, in some of the country's water ecosystems, there are several beneficiary provinces in water resources.

Also, the lack of simple methods for the restoration and rehabilitation of water bodies and the implementation of such operations requires hefty costs and complex procedures and the impossibility of proper restoration of an ecosystem as another challenge.

In Iran, water resource management has been influenced by global water management paradigms over time, and "river management" projects with structural methods are still a priority. Even past mistakes of channeling rivers are repeated under such headings as the "tourism plan". Such projects are usually implemented by municipalities or the Cultural Heritage and Tourism Organization. It can also be said that water and environmental experts are not present at the decision-making levels of these projects. The topics of domestic studies related to river restoration are limited to preliminary studies (often interval scale). For river restoration to enter the practical phase from the theoretical and study stage, river management indicators must be upgraded from the old to the new approach; That is, single-purpose goals with multi-purpose, engineering expertise with multidisciplinary expertise, cross-sectional actions with continuous actions, intermittent approaches with basin attitudes, hydraulic time scale with geomorphic time scale, short-term responsibility with long-term responsibility should be replaced, and a conservation program should be part of the planning and implementation activities.

The new approach to restoration unlike the old approach, focuses on multi-purpose goals and is a multidisciplinary specialty, and requires continuous action instead of focusing on structural and cross-sectional actions. Also, the time scale of related studies is broader than the old approach and requires more long-term responsibility. In the new approach, protection and maintenance of the implemented plan is part of the work. The comprehensive river management approach is the implementation of river management with the aim to preserve and rehabilitate the diverse ecosystem and landscape of rivers, in terms of natural processes of the river system (including erosion, transmission, and sedimentation) and maintaining the harmony of life, history and the culture of residents. It should be noted that in projects related to river restoration, according to the existing conditions and project objectives, the level of restoration can be considered. On the other hand, restoration levels show that the restoration or naturalization of the river does not only mean complete and ideal restoration, but as much as the condition of the river and its degraded environment can be improved, in fact, the level of restoration has been met.

In relation to restoration, three index references can be named^46–48^: (1) River Restoration Network Guide by (Asian River Restoration Network) ARRN in Asia (Asian River Restoration Committee), (2) RRC (Center River Restoration) Guide to River Restoration in the UK, (3) Guide to urban river basin enhancement methods by URBEM (Methods Urban River Basin Enhancement) in Germany. In this regard, the reference of the Asian River Restoration Network (ARRN) is the closest to the situation in Iran. The European Center for River Restoration (RRC) discusses resuscitation approaches and techniques in more detail. The study from this reference shows that despite the climatic differences between Iran and European countries, the approaches and implementation techniques mentioned in it are applicable in Iran and do not depend much on the climate. The Urban River Basin Enhancement Guide (URBEM) also provides relevant information, especially on urban rivers, due to its two-pronged approach to the discussion from an urban and river perspective. In total, issues related to urban river restoration can be raised in four different axes based on the ARRN guide (Table 2).

**Table 2:** Urban river restoration axes^47^.

Axis	Description
Water cycle	Water quantity (surface and groundwater), water quality, water safety and flood risk, invasion of bed and privacy
Landscape	River landscape, pleasantness and social efficiency
Culture	Stakeholders, awareness raising, building culture
Nature	Naturalization and restoration of natural river structure (morphology, erosion and sedimentation, runoff and landslides), Ecosystem restoration (biodiversity)

## Study of the restoration of rivers in the world

In this section, we take a brief look at the global experiences of river restoration. China, Japan and South Korea have a history of nearly two decades in rehabilitation and restoration. They have formed a joint database of their experiences since 2006 with the creation of the Asian River Restoration Network (ARRN), with which India, Pakistan, Malaysia, Thailand, and Australia are collaborating^47^. In Japan, the rehabilitation of the Izumi River in Yokohama over ten years (1998–1998) was one of the pioneering projects aimed at developing recreation and reconnecting the river with the environment. According to the results of this study, the attractiveness of the river shows 87%, the recurrence of the river 75%, and the improvement of wildlife 68%. In 2008, the Ministry of Land, Infrastructure, Transport, and Tourism set up a committee to revise the axis nature of river works policy. The committee's investigations led to the development of new standards for river restoration. Since 2002, the ministry has operated large-scale river basin rehabilitation projects^49^. Muhar et al. studied twenty river restoration cases in 9 countries in northern, eastern, and central Europe, which were implemented under the auspices of the project restoring rivers for effective catchment management (REFORM) and funded by the European Union. This project has implemented, standard river restoration measures, such as instream measures (such as adding slate and wood) and planform measures (such as Meander rehabilitation, river widening)^50^. The restoration of rivers in Switzerland has a history of more than three decades. From 1979 to 2012, 848 projects were implemented, and 232 of these projects were evaluated. The total length of the rehabilitated rivers is 307 km using various measures such as habitat preparation, canal reconstruction, deculverting, coastal construction, bioengineering, canal diversion, floodplain rehabilitation, flood protection, tourist management, and improving water quality^51^. The Kissimmee River Project in Florida is one of the most successful river restoration experiences in the United States. 167 km of the river was canalized to control floods in the 1960s, and since 2001, part of the main restoration waterway and flow control structures have been removed. This has resulted in increases in dissolved oxygen, reduction of the total phosphorus by 50%, and restoration of fish and bird species to the expected levels, all these factors are indicators of project success^52^.

## Analysis of restoration methods and techniques

In this section, we analyze some of the techniques used in river restoration. River restoration techniques can be divided into three general categories: hard engineering techniques (structural methods), Soft engineering techniques (no-structural methods), and watershed management (Integrated River Basin Management (IRBM))^43,53^. Hard engineering techniques are divided into two groups: longitudinal structures and transverse structures. Some of these structures are also used in the traditional method, but in river restoration, it is important to consider their role in the whole river and catchment. These methods include: Longitudinal structures (such as sealing wall, protective cover of bed and side), Transverse structures (such as water breakers, slope control structures, overflows, submerged plates). Soft engineering techniques are often environmentally friendly. In this way, they always try to organize the river naturally. The cost of these methods is lower than structural methods and they have more flexibility against natural disasters. At the same time, they are easier to manage and monitor than structural methods. In the non-structural method, the river is gradually improving and the gradual improvement of the river will lead to more stable conditions. These methods include: Non-structural cover (vegetation cover, geosynthetic cover), restoration (water storage ponds, porous paving stones, porous ponds, trenches filled with sandy materials). Table 3 shows some of the techniques used in river restoration^43,53,54^.

**Table 3:** Some techniques used in river restoration.

Technique	Description	Types		Ref
DE culverting	It is a special type of river restoration in urban areas, when it is completely covered by the waterway and has become a culvert			^56^
Dam removal	Elimination of structures that disrupt or separate the flow is one of the common measures to restorea river. If this structure is a dam, the river restoration project will be combined with the dam removal project			^53^
Large woody debris	Large wood debris are used. These structures are designed to direct the mainstream to the center of the channel	Crucifix Ground Anchoring (tree on a rope) Staking		^54^
Flow modification	Actions aimed at changing flows within a river, including changes in the volume, frequency, time, or duration of flows			^43^
Floodplain reconnection	Floodplain areas promote the flow of living things and materials between river areas and floodplains. This method increases the frequency of inundation			
Flow deflectors	Deflectors are made horizontally and vertically, and by focusing on the flow, increase its velocity locally and create areas with different sediment dispersion	D deflectors Islands Dragons teeth		^54^
Channel re-shaping	Extensive drainage activities in the past have caused the river canals to become wider and deeper and the bedrock material to be removed. Re-shaping the channel in order to restore the normal cross-section of the channel is approximate	Bank-sliding Aquatic berm Causeway		
Gravel reintroduction and enhancement	Raising the riverbed increases the flow velocity, decreases the water depth and increases the diversity of the habitat. This can be done by introducing a suitable bed on which pebbles of various sizes are located	Bed raising Enhancing existing gravel Shoals		
Protect river banks by using tree roots	It is used in rivers with sandy materials and high flow			
Brushwood mattress	They are used in rivers with clay and low flow materials			

## Conclusion

Currently, river restoration and rehabilitation is a typical response to declining river health, and its importance in managing water resources is growing. The most common reactions to river deforestation are actions aimed at maintaining the functioning of the existing ecosystem and limiting or reducing human impacts on rivers. Such activities, often under the heading of integrated water resources management, include controlling the effects of point and scattered pollutants, over-harvesting of water and development in the catchment area. In this study, various challenges of aquatic ecosystems, especially rivers, and restoration approaches were presented according to global experiences, along with different principles and techniques. Appropriate action can be taken according to the conditions in Iranian rivers, including the Zayandehroud River. Due to the novelty of the approach to river restoration in Iran, it is necessary to take full advantage of global experiences to pay attention to the differences between the conditions of these countries and Iran. Adapt as necessary the existing principles and approaches to the conditions of Iran. On the other hand, restoration projects require expertise and skills in various fields. In a revitalization project, careful planning, clear goals, and communication with all those involved in the project significantly improve the chances of achieving the goals. For river restoration to enter the practical phase from the theoretical and study stage, river management indicators must be upgraded. In the countries of the Middle East, especially Iran, it seems that the dominant paradigm of water management is still in the stage of controlling nature instead of adapting to it. The success of restoration projects requires not only a change in the attitude of managers, rules, and structures, but also a change in the attitude of all ecosystem stakeholders and the involvement of the people in all stages of the project (planning, implementation, monitoring).This change of attitude can accelerate the restoration of ecosystems with actions such as political commitment to allocate more funds, remove existing barriers, reduce the damage that failure to restoration ecosystems for economy, community.

## Data Availability

Available from the corresponding author on reasonable request.
